# Single-Nucleotide Polymorphisms in *WNT* Genes in Patients with Non-Syndromic Orofacial Clefts in a Polish Population

**DOI:** 10.3390/diagnostics14141537

**Published:** 2024-07-17

**Authors:** Alicja Zawiślak, Krzysztof Woźniak, Gianluca Tartaglia, Xabier Agirre, Satish Gupta, Beata Kawala, Anna Znamirowska-Bajowska, Katarzyna Grocholewicz, Felipe Prosper, Jan Lubiński, Anna Jakubowska

**Affiliations:** 1 Department of Interdisciplinary Dentistry, Pomeranian Medical University, 70-111 Szczecin, Poland; katarzyna.grocholewicz@pum.edu.pl; 2 Department of Maxillofacial Orthopaedics and Orthodontics, Institute of Mother and Child, 01-211 Warsaw, Poland; 3 Department of Orthodontics, Pomeranian Medical University, 70-111 Szczecin, Poland; krzysztof.wozniak@pum.edu.pl; 4 Department of Biomedical, Surgical and Dental Sciences, University of Milan, 20122 Milan, Italy; gianluca.tartaglia@unimi.it; 5 Centro de Investigación Médica Aplicada, IDISNA, Universidad de Navarra, Avenida Pío XII-55, 31008 Pamplona, Spain; xaguirre@unav.es (X.A.); fprosper@unav.es (F.P.); 6 Hereditary Cancer Center, Department of Genetics and Pathology, Pomeranian Medical University, 70-111 Szczecin, Poland; satish1482@gmail.com (S.G.); jan.lubinski@pum.edu.pl (J.L.); 7 Department of Dentofacial Orthopaedics and Orthodontics, Wrocław Medical University, 50-425 Wrocław, Poland; ortodoncja@umed.wroc.pl (B.K.); aznamirowska.b@gmail.com (A.Z.-B.); 8 Laboratory of Molecular Biology and Genetic Diagnostics, Pomeranian Medical University, 70-111 Szczecin, Poland; anna.jakubowska@pum.edu.pl

**Keywords:** cleft palate, cleft lip, birth defect, orofacial cleft, genetic variation, polymorphism, *WNT*

## Abstract

Non-syndromic orofacial cleft (OFC) is the most common facial developmental defect in the global population. The etiology of these birth defects is complex and multifactorial, involving both genetic and environmental factors. This study aimed to determine if SNPs in the *WNT* gene family (rs1533767, rs708111, rs3809857, rs7207916, rs12452064) are associated with OFCs in a Polish population. The study included 627 individuals: 209 children with OFCs and 418 healthy controls. DNA was extracted from saliva for the study group and from umbilical cord blood for the control group. Polymorphism genotyping was conducted using quantitative PCR. No statistically significant association was found between four variants and clefts, with odds ratios for rs708111 being 1.13 (CC genotype) and 0.99 (CT genotype), for rs3809857 being 1.05 (GT genotype) and 0.95 (TT genotype), for rs7207916 being 0.86 (AA genotype) and 1.29 (AG genotype) and for rs12452064 being 0.97 (AA genotype) and 1.24 (AG genotype). However, the rs1533767 polymorphism in WNT showed a statistically significant increase in OFC risk for the GG genotype (OR = 1.76, *p* < 0.001). This research shows that the rs1533767 polymorphism in the *WNT* gene is an important risk marker for OFC in the Polish population.

## 1. Introduction

Craniofacial clefts, particularly cleft lip and cleft palate (CL/CP), are the most common craniofacial birth defects in humans, posing a significant burden on both individuals and societies. Approximately 1 in 700 newborn children worldwide are born with orofacial clefts (OFCs). Its prevalence, however, varies by geographical region and ethnic origin [1]. OFCs are a heterogeneous group of anomalies encompassing face and oral cavity, that may be divided into three general types: those affecting only the lip (CL), those affecting the lip and palate (CL/P) and those affecting only the palate (CPO) [2].

As a medical condition, OFC requires multidisciplinary care in order to be successfully treated. Most patients initially experience difficulties with feeding, speech, hearing, and the oral cavity function. Even though clefts can be surgically corrected, patients often undergo multiple surgeries, including maxillofacial and dental interventions, along with speech therapy and hearing aids to be able to function properly. There is no doubt that these malformations have a significant psychological impact on patients at all stages of their lives, regardless of treatments that they receive. Birth defects are associated with a higher risk of mental health problems and a higher mortality rate at all stages of life [3].

There are a number of factors that contribute to the development of birth defects. Understanding the factors responsible for these malformations is therefore of utmost importance in order to prevent them. OFCs can either accompany congenital syndromes, in which case they are associated with genetic mutations, or they can occur separately as isolated defects. The causes of non-syndromic clefts often lie in the interaction between genetics and environmental factors [4,5]. Epigenetic processes, such as DNA methylation or histone modification, as well as non-coding microRNAs that regulate gene expression, are also influenced by environmental factors and may contribute to clefting [6,7].

Genes engaged in craniofacial development are plausible candidates for cleft occurrence. The Wnt signaling pathway is critical for proper facial growth in the embryo in an animal model. It plays a crucial role in a number of aspects of craniofacial development, such as axis formation, the survival of neural crest cells and the development of the brain [8].

Many research studies have suggested a role of the *WNT* gene family in the pathogenesis of OFCs [9,10,11,12,13]. *WNT* genes are expressed in the developing facial ectoderm. There is a link between variants in the *WNT* genes and human non-syndromic clefts. The *WNT* gene has been shown to be associated with CL/P in Europeans and Hispanics [14,15].

To investigate the role of *WNT* genes in non-syndromic orofacial clefts in the Polish population, we examined five SNPs (rs1533767, rs708111, rs3809857, rs7207916 and rs12452064) in *WNT* genes, which have been associated with CL/P in other populations.

## 2. Materials and Methods

### 2.1. Study Population

This research study was conducted on a randomly selected group of children with non-syndromic cleft lip with or without cleft palate (NSCL/P) as well as healthy controls. The study involved patients undergoing orthodontic treatment at either the Department of Orthodontics at Pomeranian Medical University in Szczecin or the Department of Dentofacial Orthopedics and Orthodontics at Wroclaw Medical University. To diagnose existing congenital defects and identify differential diagnoses for monogenic syndromes associated with NSCL/P. The NSCL/P group (*n* = 209) underwent medical and anamnesis histories, along with clinical examinations.

Clefts were classified according to the World Health Organization’s International Statistical Classification of Diseases and Related Health Problems—ICD 10, specifically under the sections Q35–Q37, which cover congenital malformations, deformations, and chromosomal abnormalities [16].

The control group consisted of 418 randomly selected patients (average age: 14.0 + 10.2 years) whose genetic material was stored in the biobank of the Department of Genetics and Pathology at Pomeranian Medical University. Both the NSCL/P patients and the control group were matched based on age and geographical location. The number of participants from Szczecin was 180 and the number of participants from Wroclaw was 238, totaling a number of 418.

### 2.2. Ethical Approval

This study was approved by the Bioethics Committee of the Pomeranian Medical University in Szczecin, in accordance with GCP—Good Clinical Practice (KB-0012/77/10). Pomeranian Medical University Ethics Committee approved the oncology biobank project (BN-001/174/05 dated 11 October 2005). Prior to participating in the study, all patients or their legal guardians provided informed consent. The control group consisted of biobank materials obtained from umbilical cord blood deposited at the Department of Genetics and Pathomorphology at Pomeranian Medical University in Szczecin. Control group individuals were selected based on their age and region of birth.

### 2.3. Samples

The group of individuals with NSCL/P provided saliva samples of 2 mL using Oragene collection kits (DNA Genotek Inc., Stittsville, ON, Canada). For 30 min before the collection of biological material, subjects were not allowed to consume solid food. The samples were stored at room temperature in a dry, light-protected place. The DNA was isolated using an automatic Chemagen set. The DNA extracted from the samples was stored in a freezer at −20 °C. In the control group, DNA from umbilical cord blood was extracted using the standard method described by Lahiri et al. [17].

### 2.4. DNA Extraction

A total volume of 0.3 mL of whole blood was mixed with 3 mL of lysis buffer, containing 0.32 M sucrose, 5 mM MgCl_2_, 1% Triton X-100, and 10 mM Tris-HCl (pH 7.5). The solution was incubated for 5 min at room temperature. The mixture was centrifuged at 2000× *g* for 5 min. The supernatant was discarded, and the pellet containing cell nuclei was retained. The pellet was resuspended in 400 μL of a solution containing 10 mM Tris-HCl (pH 8.0), 2 mM EDTA, and 400 mM NaCl. 60 μL of 10% SDS and 3 μL of 20 mg/mL proteinase K were added. The mixture was incubated for 12–18 h at 37 °C. Following this, 100 μL of 6 M NaCl was added to the mixture and mixed for 15 s. The mixture was centrifuged at 2000× *g* for 10 min. The supernatant was transferred to a new tube and 2 volumes of cold 95% ethanol were added. DNA precipitated, was collected using a pipette, washed in 70% ethanol, and dried. The dry DNA pellet was dissolved in 100–200 μL of TE buffer (10 mM Tris-HCl, 1 mM EDTA, pH 8.0).

### 2.5. Genotyping

We selected 5 SNPs in 3 *WNT* genes that have been previously suggested as candidate genes for cleft/lip palate based on studies with animal models or association studies in humans [18,19,20,21,22] to test for association with orofacial clefts in the polish population. Details of the selected SNPs and genes investigated are presented in Table 1.

Genotyping of rs1533767, rs708111, rs3809857, rs7207916 and rs12452064 was performed using the real-time PCR-based TaqMan technique with LightCycler 480 II (Roche Diagnostics, Basel, Switzerland). The mixture (5 μL total) consisted of 2.5 μL LightCycler 480 Probes Master Mix (Roche Diagnostics), 0.0625 μL of each SNP TaqMan Genotyping Assay × 40 (Applied Biosystems, Waltham, MA, USA), 1 μL DNA (25 ng/μL) and 1.4375 μL deionized water (Roche Diagnostics). The PCR conditions were previously described by Zawiślak et al. [23]. On each plate, four negative controls without DNA were included to monitor potential contamination.

### 2.6. Statistical Analysis

A logistic regression test for nonlinear analysis was used for statistical analysis to assess the effect of independent variables on the dichotomous dependent variable.

We have chosen logistic regression for our analysis due to its suitability for binary outcomes such as cleft palate presence, ability to handle multiple SNPs, and capability to adjust for confounding variables. It provides interpretable odds ratios, accommodates nonlinear relationships inherent in genetic data, and performs well with our adequate sample size (209 cases, 418 controls) and SNP minor allele frequency (>0.15). This method ensures a rigorous and comprehensive analysis of our genetic data, delivering clear insights into the association between SNPs and OFC risk.

The odds ratio (OR) with 95% confidence interval (95% CI) was calculated to estimate the risk of a craniofacial cleft defect. The most common genotype was used as a reference. The occurrence of each genetic variation was compared to the control group in order to assess the risk carried by the genotype on the development of the birth defect. The significance of individual logistic regression coefficients was evaluated using Wald’s test. The orofacial cleft risk was assessed for each genotype. The significance of particular logistic regression rates was assessed using the Wald’s test. Statistica 10.0 (StatSoft, Tulsa, OK, USA) and R 3.0.2 (The R Foundation for Statistical Computing) were used for statistical analysis. *p*-values of less than 0.05 were considered significant.

## 3. Results

The study group consisted of 209 participants with OFC (age range, 4–30 years; mean age 17.4 ± 13.6 years). A total of 418 subjects were randomly assigned to the control group based on their age and place of birth. In both groups, relatives in the ascending line up to the second generation were Polish. The OFC group consisted of 91 women (43.5%) and 118 men (56.5%). A unilateral cleft of the lip and the hard palate was observed in 113 cases (54.1%), a bilateral cleft in 45 cases (21.5%), a cleft palate only in 32 cases (15.3%) and an isolated cleft lip in 19 cases (9.1%).

Table 2 summarizes the results obtained for selected genetic variants in *WNT* genes.

Statistical analysis shows that the presence of the rs1533767 gene polymorphism is a significantly predictive factor for the occurrence of cleft lip and palate (*p* < 0.001). The risk of cleft and palate increases in the presence of the AG genotype [OR = 1.76 (1.23–2.52)]. The result for the AA genotype was not statistically significant [OR = 0 (0-Inf), *p* = 0.977].

The rs708111 polymorphism is not a statistically significant predictive factor for the risk of orofacial cleft (*p* = 0.851). In the presence of the CC genotype, the odds ratio was 1.13 (0.69–1.85) with *p* = 0.629, while in the presence of the CT genotype, the odds ratio was 0.99 (0.66–1.50) with *p* = 0.995.

Similarly, rs3809857 does not significantly predict the risk of cleft lip and palate (*p* = 0.93). In this case, the assessment of odds ratio indicated an increased possibility of cleft lip and palate with the presence of the GT genotype [OR = 1.05 (0.72–1.54)], and a decreased possibility with the presence of the TT genotype [OR = 0.95 (0.52–1.73)]. However, both results were not statistically significant, reaching the levels of significance of *p* = 0.786 and *p* = 0.875, respectively.

The polymorphism rs7207916 was also not defined as a statistically significant predictive factor for the occurrence of cleft lip and palate (*p* = 0.214). The odds ratios for the AA and AG genotypes were 0.86 (0.5–1.47) with a significance level of *p* = 0.577, and 1.29 (0.87–1.92) with a significance level of *p* = 0.204, respectively.

Upon analyzing the risk of cleft lip and palate associated with the presence of the rs12452064 polymorphism, no statistically significant results were obtained (*p* = 0.458). In the case of the AA genotype, the risk was slightly lower [OR = 0.97 (0.58–1.62), *p* = 0.895], compared to the AG genotype where the risk increased [OR = 1.24 (0.81–1.87), *p* = 0.321]. However, these results did not find confirmation in the statistics.

**Figure 1:**
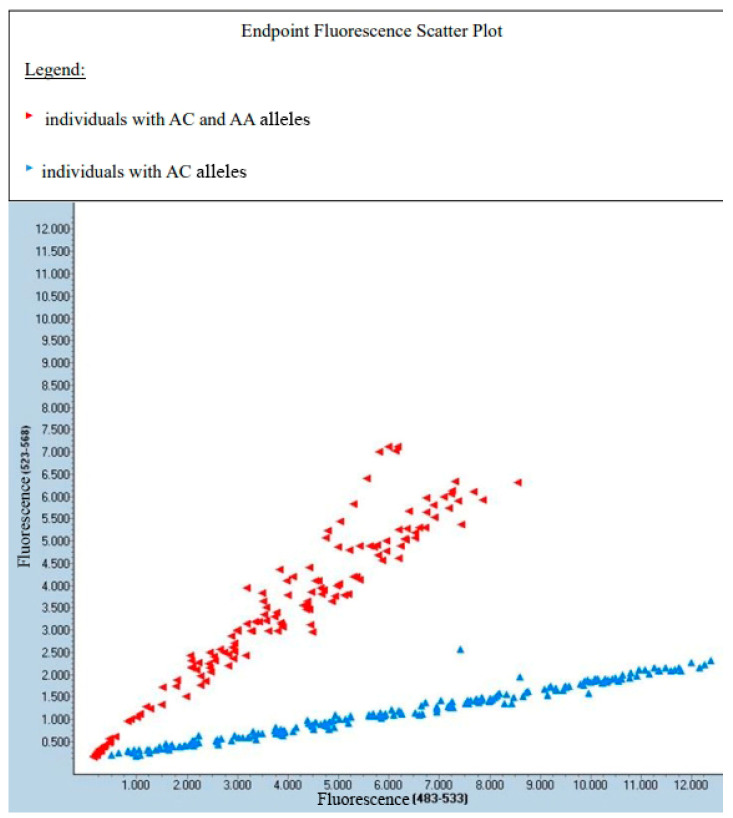
Graphical distribution of genotypes for rs1533767. Blue points represent patients with AC alleles and red points represent patients with AC and AA alleles. No AA-only alleles present.

The distribution of genotypes for rs1533767, for which *p*-value was <0.001, is shown in Figure 1 using Endpoint Fluorescence Scatter Plot, which is a graphical representation of research outcomes. It displays data points where each point represents the fluorescence intensity at the endpoint of a reaction. The *x*-axis represents the fluorescence of one allele, and the *y*-axis represents the corresponding fluorescence of a second allele. Each data point on the plot corresponds to a specific sample. The scatter plot visualizes the distribution of different samples.

The presented result of the fluorescence analysis of the sample was performed using a LightCycer 480II (Roche Diagnostics). The blue color represents the AC allele (stained with VIC dye), the green color represents the AA allele (stained with FAM dye), but in which case was not present, and the red color represents both alleles.

The results of the logistic analysis determining the risk of specific phenotypic forms of cleft defects for the studied SNPs are presented in Table 3.

Upon analyzing the results obtained for genetic variations that significantly determine the risk of OFCs, we can observe the following:

1. The presence of the AG genotype in the rs1533767 polymorphism is associated with a lower risk of developing a cleft palate with unilateral cleft lip (OR = 0.59, *p* < 0.001) compared to other observed phenotypes of the clefts.
2. The presence of the AA genotype in rs223371 reduces the risk of developing a cleft palate with unilateral cleft lip (OR = 0.33, *p* < 0.001). Conversely, the AC genotype decreases the risk of both cleft palate with unilateral cleft lip (OR = 0.26, *p* < 0.05) and cleft lip (OR = 0.08, *p* < 0.01) compared to other types of defects.

## 4. Discussion

It is a well-known fact that *WNT* genes are widely involved in regulating facial processes development as well as upper lip fusion [23]. The *WNT* gene family encodes a huge group of secreted glycoproteins, which are involved in prenatal midface development and lip fusion [24,25].

Through the binding of *WNT* ligands and receptors, a cascade of intracellular signaling pathways are activated. *WNT* product is present in the upper lip as well as hard and soft palates [26]. *WNT3*, which is mapped to chromosome 17q21.31-q21.32, is an especially vital member of the WNT gene family [27] and is required in the earliest phases of prenatal craniofacial development [18,28,29].

The ongoing advancements in research on the etiology of OFCs have not contraindicated the thesis presented in this paper regarding the multifactorial nature, but instead have reinforced the longstanding belief. Consequently, scientists have directed their focus not only towards relatively well-known environmental factors but primarily towards genetic factors underlying these developmental defects. However, despite numerous studies, many questions remain unanswered regarding the specific models and etiology of cleft lip and palate formation.

In our research, the rs1533767 polymorphism has shown an association with OFCs within a Polish population. Unfortunately, to date, only one publication has discussed this variant in relation to the co-occurrence of cleft lip and palate. This same research has identified several other single-nucleotide polymorphisms within the *WNT* gene family. However, rs1533767 exhibited the strongest correlation in a diverse population in the United States, including individuals of both Latino and European descent. Upon further analysis, a notably stronger association was observed within the European subgroup. Notably, this polymorphism resides within an exon, a coding region of the chromosome, which underscores its potential significance for future investigations [19].

Our research results did not confirm the association of the rs708111 polymorphism within the *WNT3A* gene with cleft defects. However, such an association was confirmed in the previously cited studies by Chiquet et al. [19] conducted in an American population.

The subsequent genetic changes studied in our research focused on polymorphisms rs3809857, rs7207916 and rs12452064 located in the *WNT3* gene. Unfortunately, we were unable to confirm the hypothesis proposed by Mostowska et al. [20], which suggested that the risk of isolated orofacial cleft defects nearly halved in patients with the TT allele in rs3809857. Mostowska et al. also reported statistically significant results for haplotypes rs12452064, rs7207916 and rs3809857, with *p*-values of 0.0034 and 0.0014, respectively. Their study included 210 Polish patients with isolated cleft lip with or without cleft palate, which was almost equal to our research [20]. Differences in results may arise from several factors. First, the genetic makeup of our study population might differ from Mostowska et al.’s population. However, there are no ethnic, geographical, or demographic differences that can affect allele and haplotype distributions. Additionally, other genetic or environmental factors not accounted for in our studies might influence the risk of orofacial clefts. The complex interplay between genes and environmental factors can lead to varying results depending on the population and research conditions. Although we did not confirm Mostowska et al.’s hypothesis, these discrepancies highlight the multifactorial nature of the genetic basis for OFCs.

Further studies conducted on non-Caucasian populations investigated the potential association between 13 SNPs located in *WNT*,* WNT5A*,* WNT3A*,* WNT8A*,* WNT11* and *WNT9B* genes in OFCs. Is has been proven that *WNT3* rs142167 and rs9890413 significantly increased the risk of OFCs [15], whereas in research investigating the impact of *WNT3* rs3809857 and rs9890413 SNPs on the risk of OFCs, the authors found that the rs3809857 variant significantly decreased the risk of malformations [18]. These findings indicate the genetic diversity underlying OFCs across different populations. The significant association of *WNT3* rs142167 and rs9890413 with increased OFC risk in non-Caucasian populations suggests that certain SNPs may have population-specific effects. The study showing that rs3809857 reduces the risk of OFC highlights the complexity of genetic interplay and potential protective roles of specific variants.

The meta-analysis by Wang B. et al. [21] included four studies on the rs3809857 polymorphism and OFC patients, covering 679 cases and 976 controls [20,22,30,31]. As no heterogeneity was observed, a fixed-effect model was employed for calculating the association between SNP and OFCs, revealing a significant link between rs3809857 and OFC risk (G/T, OR = 1.34, 95% CI: 1.15–1.56, *p* = 0.0001). These findings robustly demonstrate rs3809857’s role in increasing OFC susceptibility across diverse study populations. The consistent effect size (OR = 1.34) highlights a 34% increased risk associated with this SNP. This underscores the relevance of genetic screenings in clinical assessments for OFC risk management and potential targeted therapies.

It should be emphasized that two of the polymorphisms included in our studies were verified for the first time based on the native population. In the available literature, regarding the assessment of the risk of isolated orofacial clefts in a Polish population, reports can only be found for three of the five single-nucleotide polymorphisms examined in our studies: rs3809857, rs12452064 and rs7207916.

The existing reports on the predisposition of genetic changes to isolated cleft defects are usually based on single studies and are not consistent with studies conducted in other scientific centers dedicated for this research field. Often, these studies are based on ethnically distinct populations from the Polish population, which constitutes another difficulty.

## 5. Conclusions

The results of our study suggest that the *WNT* gene rs1533767 SNP is associated with an increased risk of non-syndromic orofacial clefts in the Polish population. Our findings highlight the potential role of the *WNT* signaling pathway in craniofacial development and suggest that the rs1533767 SNP could be a genetic marker for increased susceptibility.

Nevertheless, it is important to note that while our findings indicate a significant association, they do not imply an absolute risk. Genetic susceptibility is only one aspect of the multifactorial nature of these congenital defects. Environmental factors, interactions between multiple genes, and other epigenetic mechanisms may play critical roles in their development as well. Future studies with larger sample sizes and diverse populations are necessary to validate these findings and further explore the complex interplay of genetic and environmental factors.

## Figures and Tables

**Table 1:** Candidate genes and SNPs studied.

SNP	Gene	Chromosome	Base Pair Position	Base Change	SNP Type	MAF
rs1533767	*WNT11*	11	75,905,800	A/G	exon	0.22 (A)
rs708111	*WNT3A*	1	228,191,365	C/T	5′ upstream	0.53 (C)
rs3809857	*WNT3*	17	46,770,948	G/T	intron	0.30 (G)
rs7207916	*WNT3*	17	46,801,984	G/A	intron	0.59 (G)
rs12452064	*WNT3*	17	46,790,821	G/A	intron	0.42 (G)

MAF—Minor Allel Frequency; A—adenine; C—cytosine; G—guanine; T—thymine.

**Table 2:** OFC risk and polymorphisms in *WNT* genes (namely rs1533767, rs708111, rs3809857, rs7207916, rs12452064).

Genotype	OR (95%CI)	*p* (Wald’s Test)	*p* (LR-Test)
rs1533767 ref.=GG			<0.001
AA	0 (0-Inf)	0.977	
GG	1.76 (1.23–2.52)	0.002	
rs708111 ref.=TT			0.851
CC	1.13 (0.69–1.85)	0.629	
CT	0.99 (0.66–1.50)	0.995	
rs3809857 ref.=GG			0.93
GT	1.05 (0.72–1.54)	0.786	
TT	0.95 (0.52–1.73)	0.875	
rs7207916 ref.=GG			0.214
AA	0.86 (0.5–1.47)	0.577	
AG	1.29 (0.87–1.92)	0.204	
rs12452064 ref.=GG			0.458
AA	0.97 (0.58–1.62)	0.895	
AG	1.24 (0.81–1.87)	0.321	

**Table 3:** Risk of specific phenotypic forms of OFCs for studied SNPs in WNT gene.

SNP	Genotype	Type of Cleft	OR (95%CI)	*p*-Value
rs1533767 ref.=CPO	AG	CL	0.5 (0.15–1.58)	<1
		CL/P (bilateral)	0.63 (0.25, 1.61)	<1
		CL/P (unilateral)	0.59 (0.26, 1.35)	<0.001
	AA	CL	0 (0-Inf)	<1
		CL/P (bilateral)	0 (0-Inf)	<1
		CL/P (unilateral)	0 (0-Inf)	<1
rs708111 ref.=CPO	CC	CL	0.27 (0.04, 1.85)	<1
		CL/P (bilateral)	0.29 (0.07, 1.14)	<0.05
		CL/P (unilateral)	0.6 (0.19, 1.91)	<0.001
	CT	CL	0.78 (0.19, 3.18)	<1
		CL/P (bilateral)	0.49 (0.16, 1.5)	<0.05
		CL/P (unilateral)	0.53 (0.19, 1.49)	<0.001
rs3809857 ref.=CPO	GT	CL	0.98 (0.26, 3.64)	<1
		CL/P (bilateral)	1.05 (0.37, 2.95)	<1
		CL/P (unilateral)	0.77 (0.32, 1.84)	<0.001
	TT	CL	0.92 (0.07, 12.31)	<1
		CL/P (bilateral)	2.75 (0.47, 15.95)	<1
		CL/P (unilateral)	1.34 (0.26, 6.96)	<0.001
rs7207916 ref.=CPO	AA	CL	1.6 (0.2, 12.69)	<1
		CL/P (bilateral)	1.33 (0.26, 6.74)	<1
		CL/P (unilateral)	2.12 (0.53, 8.58)	<0.01
	AG	CL	1.29 (0.32, 5.19)	<1
		CL/P (bilateral)	1.23 (0.44, 3.45)	<1
		CL/P (unilateral)	1.59 (0.65, 3.89)	<0.01
rs12452064 ref.=CPO	AA	CL	0.21 (0.02, 2.48)	<1
		CL/P (bilateral)	0.64 (0.16, 2.58)	<1
		CL/P (unilateral)	0.58 (0.17, 2)	<0.001
	AG	CL	0.9 (0.2, 4.08)	<1
		CL/P (bilateral)	0.6 (0.18, 1.98)	<1
		CL/P (unilateral)	0.77 (0.27, 2.21)	<0.001

## Data Availability

All data are available from the corresponding author upon request.
